# The CROPROTECT project and wider opportunities to improve farm productivity through web‐based knowledge exchange

**DOI:** 10.1002/fes3.80

**Published:** 2016-03-06

**Authors:** Toby J. A. Bruce

**Affiliations:** ^1^ Rothamsted ResearchHarpendenHertfordshireAL5 2JQUK

**Keywords:** Farmer research networks, impact, innovation, research policy, sustainable intensification, translational research

## Abstract

A key global 21st century challenge is to maximize agricultural production while minimizing use of resources such as land, water, and energy to meet rising demand for produce. To meet this challenge, while also adapting to climate change, agriculture will have to become more knowledge intensive and deploy smarter farming techniques. The intention of this study was to: (1) Highlight the opportunity for web‐based knowledge exchange to increase farm productivity and thus contribute to achieving food and energy security, (2) Give some examples of online farming information services such as the “CROPROTECT” tool I am developing in the UK, the CABI “Plantwise” Knowledge Bank and the IRRI “Rice Doctor,” and (3) Consider lessons learnt so far. There are huge opportunities to facilitate knowledge exchange through online systems for farmers and people who advise farmers. CROPROTECT is interacting with users to determine priorities in terms of the pests, weeds, and diseases covered and is providing key information to assist with their management. Knowledge is a critical input for farming systems. Crop protection in particular is becoming more difficult due to evolution of pest resistance and changes in legislation. Up to date information can be made rapidly available and shared online through websites and smartphone Apps. Agricultural extension no longer relies solely on physical meetings and printed documents. The capacity to share information via the Internet is tremendous with its potential to reach a wide audience in the farming community, to provide rapid updates and to interact more with the users. However, in an era of information deluge, accessing relevant information and ensuring reliability are essential considerations. There is also a need to bring science and farming communities together to turn information into relevant farming knowledge.

## Introduction

Agriculture is facing huge pressures in the 21st century to produce more as demand rises but, in an increasingly resource constrained world, there is less scope for it to use more resources such as land, water, energy, and nutrients (Baulcombe et al. 2009). The challenges of meeting surging demand for food, water, and energy while also adapting to climate change were clearly explained by Sir John Beddington in his “Perfect Storm” speech (Beddington 2009). The improvement of advisory services was identified as a priority in the UK government Foresight report, “The Future of Food and Farming” (Foresight 2011) and lack of information flow between scientists, practitioners, and policymakers is mentioned by Pretty et al. (2010) as a constraint to achieving growth in food production. Farm businesses need to find smart ways of producing and protecting high value and high yield produce. To achieve sustainable intensification of agriculture, there is a need to improve access to information to allow farmers and others in the farming community to make better decisions.

Effective knowledge exchange allows farmers and agronomists to benefit from scientific and technical advances. It can also provide invaluable feedback from farmers to the research community that will help improve the research and make it better targeted to farmers' needs and thus increase its impact (Phillipson et al. 2012; Kindred 2015). However, unfortunately, the prospect of stakeholder engagement is sometimes viewed as a distraction by the scientific community (Phillipson et al. 2012) and linkages between the advisory system and the scientific system may need improvement (Klerkx and Proctor 2013). Sometimes the solutions that are appropriate may not be immediately apparent either to the land managers or to the researchers and interaction can help to generate novel solutions. In terms of information about potential solutions, farmers and agronomists require decision support but not decision making because they are the ones to decide what is most appropriate for their local conditions (Wood et al. 2014).

For exploitation of research findings, current thinking is moving from a model of knowledge transfer (Garforth et al. 2004) toward a model of knowledge exchange (Phillipson et al. 2012; Ward et al. 2012; Wood et al. 2014). The implication of “knowledge transfer” is that it is separate from knowledge production but if this separation is not accepted, and networks allow transfer in both directions, then it is possible to have a more interactive model of knowledge production (Phillipson et al. 2012). This means that farmers and agronomists are more involved and provide input and feedback into the process. The book “Farmer First” by Chambers et al. (1989) radically prioritized farmer participation in development of knowledge but since then there has been growing realization that a wider innovation system is needed and the farmer cannot be considered in isolation (Pretty and Chambers 1994; Scoones and Thompson 1994, 2009; Klerkx et al. 2012; Wood et al. 2014). Thus, two‐way flow of information can benefit all parties involved.

There is increasing awareness of the important role played by farmer‐to‐farmer dissemination pathways for effective sharing. Scientists create knowledge but farmers decide what is best practice (Kindred 2015). Farmers and agronomists are more likely to be convinced if they see a neighboring farmer successfully using a new technique or seeing it demonstrated at a trial site than reading about it. For example, farmer‐to‐farmer networks have played a key role in successful adoption and upscaling of a push‐pull companion cropping system developed by *icipe* and Rothamsted, which is now used by approximately 100,000 smallholder farmers in East Africa (Amudavi et al. 2009). Although farming systems are different in different parts of the world, they all need information to optimize farm productivity. Whether in the developing or developed world, growers value real farming experience and knowledge of the particular over knowledge in general (Wood et al. 2014). Wood et al. (2014) found that New Zealand farmers seek knowledge that can be applied to their individual farm by contacting individuals who can share the experiences of other equally individual farms. Mechanisms need to be found to couple these dissemination pathways with access to science based knowledge and data to improve farming operations. There is potential to do this through online knowledge sharing provided that there are people and structures set up to provide the relevant content.

## Online Knowledge Sharing Platforms

There are opportunities to develop online systems for knowledge exchange. Although they will never be a complete substitute for face‐to‐face meetings, they can be highly complementary, especially in situations where face‐to‐face meeting do not occur often. Indeed, rather than replacing meetings in person they can open lines of communication that lead to subsequent new contacts and face‐to‐face meetings. Online systems have major advantages of improved accessibility, and mean that there can be a wider reach while saving time and travel costs. Internet access is widespread and increasingly via mobile devices which mean that information posted can be consumed at a time and place convenient for the user. Furthermore, electronic materials can be rapidly updated and allow interaction with users. Exciting developments in online publishing and social media have occurred but for improved knowledge exchange better systems integrating formal scientific reporting and informal farmer knowledge networks are needed.

**Figure 1:**
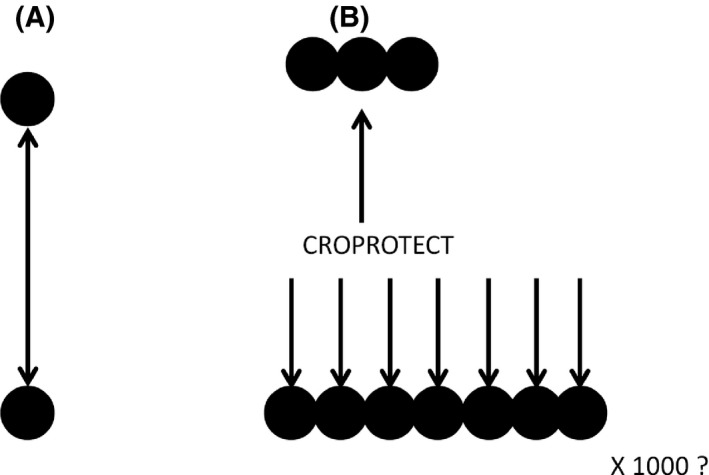
Interaction between knowledge brokers and users. (A) Driving to a farm to meet in person allows an in depth conversation but means farms have to be visited one at a time. (B) Providing online information is a more faceless form of communication but has the advantage of being able to reach a large number of users.

There is immense potential to share information about “what works” via the Internet because of its tremendous reach and capacity for sharing information (Fig. 1). The way information is structured, organized, and delivered needs consideration so that it can be optimized. Systems need to be developed to translate research findings into summaries of what they mean for the farmer. There is extensive information available online but it is scattered and of varying quality. The challenge is to find relevant information and provide it in a format where the key points are clearly explained. Some examples of online farming information services include:

### Plantwise Knowledge Bank

The CABI online Plantwise Knowledge Bank, www.plantwise.org/KnowledgeBank, provides pest identification tools and a database of online factsheets about plant health (Leach and Hobbs 2013). It is used by “Plant Doctors” who provide extension advice to farmers in developing countries. This is a large scale operation with 3500 plant doctors in 1500 plant clinics in 34 countries. There is a Plantwise Factsheets Library App for Android smartphones as well as the website. The Plantwise program provides actionable knowledge to farmers through community based plant clinics, encouraging exchange of information between farmers and other stakeholders and by drawing together information and making it available in a central resource – the Knowledge Bank. The combination of online information with large numbers of plant doctors to advise farmers perhaps gives the best of both online and face‐to‐face meetings, however, even the large number of plant doctors may not meet demand. The knowledge bank also contains “Factsheets for Farmers,” written by extension workers in‐country to ensure they are locally relevant and these could allow direct access by farmers who have a smartphone but not a local plant doctor, Feedback from a user survey indicated that they found there was too much information and they wanted it filtered more, they wanted more images for identification and less technical language and better links to management information (Leach and Hobbs 2013), Internet access varied from country to country and in some countries printed factsheets were used due to lack of Internet connectivity. The Plantwise Knowledge Bank continues to grow and is focusing on adding more locally relevant content (Leach and Hobbs 2013). Some factsheets may require updating. CABI is also developing “Direct2Farm” – a service that turns factsheets into short SMS and voice messages that are delivered straight to smallholder farmers via mobile phones.

### IRRI rice knowledge bank

“Rice Doctor” from the IRRI rice knowledge bank (http://www.knowledgebank.irri.org/) provides diagnostic tools and factsheets for management of rice diseases. It is available as a smartphone App for Android or iOS. “Crop Manager” provides rice, maize, and wheat farmers with personalized crop and nutrient management guidelines. Weed identification tools and information about rice crop management at different growth stages are also provided.

### PlantVillage

PlantVillage Images, https://www.plantvillage.org/, is an open access database of 50,000+ images of healthy and diseased crops. PlantVillage are launching and growing this database in order to enable the development of open access machine learning algorithms that can accurately classify crop diseases on a smartphone. It also has a function where users can ask questions via the website.

### Agriculture and Horticulture Development Board

This UK levy board has a website, http://www.ahdb.org.uk/, containing many valuable factsheets. These are in the crop management section of its website. Information is provided about field drainage, crop diseases, weed management, pest management, nutrient management, soil management, grain storage and sampling, precision farming, and stewardship.

Please note the above examples are not an exhaustive list of all online agricultural knowledge exchange systems – they are only intended to give an idea of the existing state of the art. I now provide a description of the CROPROTECT system I am developing.

### CROPROTECT

**Figure 2:**
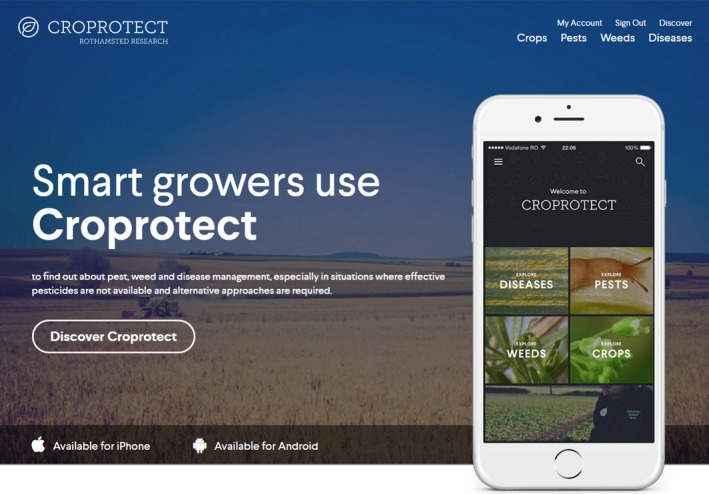
The CROPROTECT homepage (from https://croprotect.com/).

CROPROTECT is a new UK‐based website, www.croprotect.com, which we are developing to provide information about pest, weed, and disease management (Fig. 2). It is a new and growing resource for farmers and agronomists designed to provide easy access to management recommendations for key pests. From the onset we have been engaged in two‐way communication with users. This way we can build a system which is relevant to them and meets their requirements, raise awareness and grow the user base at the same time as developing the system. The first version of the website was launched when the project began in November 2014. On this, pioneer users could register and say what their pest, weed, and disease priorities were. Thus, the target pests, for which management information is being compiled, are the ones specified by the users. Not surprisingly, the most frequently mentioned targets are ones which have evolved resistance to pesticides and for which alternative or supplementary management is required. Agriculture is becoming more difficult as the era of cheap effective chemical pesticides is ending and new biological solutions and information about how to implement those solutions is required (Hillocks 2012; Andersons Centre 2014). To address these issues, CROPROTECT aims to draw together existing and new information about crop protection as there is a general problem of information sources being fragmented and disconnected (Klerkx and Proctor 2013). Alternative approaches are often more complicated relying on a combination of resistant cultivars, biocontrol, agronomic practices and rationalized, better targeted pesticide use. Information about integrated pest, weed, and disease management is scattered in disparate places which are hard for busy farmers to track down for every pest, weed, and disease threat they face.

The first pest management information pages on the website were released in February 2015. Information pages were only available to registered users to provide an incentive for users to register and specify their crop protection targets. However, we realized that registration and login was a barrier to entry and after more information pages were compiled we decided to make the information pages available to nonregistered users. At the time of writing we now have over 640 users and in the last month there were 379 visitors to the website. The system is evolving and has only been running for 1 year so far. Electronic documents can be easily updated and tweaked as new information, for example, about a new crop variety or treatment becomes available. This is a major advantage compared to printed documents which are fixed once printed.

In response to user demand for even easier access, a CROPROTECT smartphone App (for Android and iOS) was developed and launched in December 2015. Part of the inspiration for CROPROTECT was the active community of farmers, agronomists and other agricultural professionals who use social media, particularly Twitter, for farming discussions. AgriChatUK, an online farming forum, hold regular weekly discussion sessions every Thursday evening and CROPROTECT has featured in several of them. The discussions are run on Twitter using the #agrichatuk hashtag. They reach many people in the agricultural sector and the AgriChatUK Twitter account has over 18,000 followers. We are using these channels to raise awareness of CROPROTECT and have a representative of, AgriChatUK, on the steering committee for CROPROTECT. Guidance is also being obtained from the Agriculture and Horticulture Development Board (AHDB, a UK levy board), the National Farmers Union and three agronomist organizations (Hutchinsons, Agrii, and AICC).

While the primary aim of CROPROTECT is to provide management recommendations, it also contains a reporting function through which users can say what their pest targets are. User reports are mapped on a GIS (geographical information system) using Google maps which is used to create pest incidence maps. These provide crowdsourced information on which pest, weed, and disease problems occur in different parts of the country. As the number of users grows there may be further opportunities to use these data to predict the spread of pests, weeds, and diseases and could be particularly powerful if coupled with meteorological data and soil data. User data are kept are not put in the public domain unless they are anonymized to protect user privacy. The resolution of pest, weed, and disease incidence maps provided online is restricted so that individual farms are not identified.

Feedback from users has been very positive. They appreciate having straightforward information focusing on key management recommendations. We have emphasized quality and relevance of information over quantity because we know that users' time is limited and they require key pieces of information rather than to have to sift through many pages. The ability to link to other information resources is expected to make CROPROTECT even more useful. We are currently linking extensively to AHDB resources. The capacity to share information via the Internet is tremendous and access is increasingly via mobile devices. These have the potential to reach a wide audience in the farming community, to provide rapid updates and to interact more with the users. In the Internet age, availability of information is not the main constraint, there is more of an issue of accessing relevant information.

The UK, as a member state of the EU, is obliged to implement EU directive 2009‐128, the “Sustainable Use Directive” (European Parliament 2009), which not only places restrictions on the use of conventional toxic pesticides but also has a requirement for “Promoting the use of IPM (integrated pest management) and of alternative approaches”. IPM is where a combination of control tactics is used against pests such as biocontrol or resistant crop varieties so that reliance on pesticides is reduced (Kogan 1998). For example, resistant varieties and a decision support system using data from pheromone traps have allowed reductions in use of chlorpyrifos against orange wheat blossom midge (Bruce and Smart 2009). CROPROTECT is designed to make information about IPM more readily accessible either through the website or through the App. As such, it will put the UK in a better position for achieving the intention of EU directive 2009‐128 which is supposed to provide IPM solutions.

A major advantage of electronic systems is that they can be easily and rapidly updated as new information becomes available. They do not have to be reprinted each time there is an update and this means small changes can be made frequently. However, to ensure the information is kept up to date it is important that there is someone to maintain the site and that regular review points are set.

## Closing Yield Gaps

Scientific and technical advances need to be taken to the farmer. In addition to ensuring farmers and agronomists have access to information about crop protection to minimize crop losses in the UK, there are wider opportunities to reach the farming community globally. There are large parts of the world where average crop yields are below their climatic potential which is known a “yield gap” and where yields could improve with different land management practices (Licker et al. 2010). There are opportunities to increase yields across many parts of Africa, Latin America and Eastern Europe (Foley et al. 2011). Part of the reason for comparatively low agricultural productivity is that knowledge about improved farming practices has not yet been delivered and web‐based knowledge exchange systems could help reach areas where conventional agricultural advisory services have not reached. For example, the remote locations, small sizes and very large number of smallholder farms in Africa mean that knowledge exchange is an immense challenge. Even if there were large numbers of extension officers it would be difficult to reach all the people requiring help. The reality is that extension services are minimal and in many places nonexistent. Therefore, new methods of reaching farmers need to be found. Being successful in agriculture is challenging even at the best of times but smallholder farmers in sub‐Saharan Africa face severe constraints to their production systems due to economic disadvantage, increasingly unpredictable weather patterns, lack of inputs, and lack of extension support. Nevertheless, there is a vast potential for improvement with typical cereal yields of only 1 ton per hectare. Given the right support there is tremendous potential for yield improvement which can make big changes to the livelihoods of smallholder farmers.

In the developed world there may not be such large yield gaps but farmers are coming under increasing pressure to comply with new legislation. Knowledge sharing networks are particularly important in the area of crop protection where pesticides are no longer a reliable solution as pests are evolving resistance to old products and fewer new products are coming through the regulatory system. This is discussed in the next section.

## How to Increase Participation of Farmers, Agronomists and Researchers in Online Knowledge Sharing?

For online knowledge sharing to happen the people involved need to actively participate. Access to information technology is a prerequisite but is not as much as of a limiting factor as it was 10 or even 5 years ago. Internet use is becoming almost ubiquitous although a minority of mostly older people in developed countries or poorer people in developing countries may not take part because they do not have the technology. A Defra survey conducted in 2012 (Defra, 2013) found that 86% of UK farms had access to a computer and 29% of UK farmers had a smartphone. Even smallholder farmers in developing countries have widespread mobile phone use (Kikulwe et al. 2014) and they are increasingly using basic version of smartphones that have Internet access. The reasons why people may or may not engage in online knowledge sharing are complex but fall into a few main categories. First of all, the people involved need to know the opportunity exists. For this marketing may be needed to raise awareness. To engage some people may take part for altruistic or social reasons but to dedicate time users need to see some kind of advantage or benefit. Farmer and agronomists would check if the information is relevant to their needs and useful but also form a value judgment if the information is reliable. To raise awareness and ensure the system meets users' requirements CROPROTECT has been working with a group of pioneer farmers and agronomists right from the start of the project. Researchers need to be incentivized to provide information for online systems connecting to end users. Academic career progression is based on publication in scientific journals especially if these articles are referred to by other academic researchers and there is less reward for engagement with other beneficiaries although this is changing with many sponsors increasingly requiring academics to demonstrate “pathways to impact” for their research. Finally systems need to be accessible and user friendly and have high‐quality relevant content presented in an attractive way. Something that is difficult to access or make sense of will not be used so much. Many people spend time on social media because they enjoy the interaction and the content. Agricultural knowledge systems need to be developed so that they are a pleasure and not a pain to use.

## Lessons Learnt So Far

Online knowledge sharing systems bridging research and user communities are probably in their infancy and will develop rapidly in the next decade. General lessons and guiding principles that have been learnt so far fall into three categories of relevance, ease of use and marketing:

### Relevance

- Provide content which the users find relevant and useful
- Make information meaningful
- Tackle the research – user gap
- Provide local contextualization – tackle spatial/temporal variation
- Provide up to date information

### Ease of use

- Have a user friendly interface
- Do not make it more complicated than necessary – keep it simple
- Provide ongoing support
- Make key information easy to access and find

### Marketing

- Make sure users are aware that the system exists
- Address legislation farmers have to comply with
- Incentive to use (e.g., compliance with legislation)
- Bring farmers into design

## Conclusions

The role of communication in agricultural innovation systems deserves more attention (Leeuwis and Aarts 2011). Knowledge exchange is vital for innovation (Cooke 2001) and innovation is vital for improving agricultural productivity (Alston 2010). While sharing technical information can help we must remember that the final decision about land management practices is with the famer, however, farmer decision making is likely to be improved when the farmer is better informed. Given these requirements and the need to improve agricultural productivity in a sustainable way, there is a policy debate about how to stimulate adequate advisory systems and bridge the gap between people separated spatially and in different organizations (Klerkx and Proctor 2013). The Internet was originally invented for information exchange in a military context but has expanded into many diverse applications and is continuing to evolve (Leiner et al. 2009). Defined as, “a world‐wide broadcasting capability, a mechanism for information dissemination, and a medium for collaboration and interaction between individuals and their computers without regard for geographic location” (Leiner et al. 2009), it is easy to see that online tools could help solve 21st century agricultural knowledge exchange challenges.

The capacity exists but the computers will not do it by themselves and people are needed to imagine, develop, and use the systems. There is a need for “knowledge brokers” that can span different areas as has been pointed out earlier even before the emergence of online systems (Gibbons 1994). Ideally hybrid systems coupling the individual interactions of social media with the quality control of scientific papers that filter out the relevant information for each farm is needed. Also, for application of agricultural knowledge there does need to be “regard for geographic location” and, as pointed out by Wood et al. (2014), farmers seek knowledge that can be applied to their individual farm and are concerned with particular rather than general findings. The farmer will make decisions based on her or his knowledge of the local conditions supported by information received from external sources. Farmer confidence is important and trusted sources of online content need to be established. To promote systems which translate and share the latest research developments incentives need to be found to engage researchers in the process, perhaps for them to demonstrate their pathway to impact. Finally, knowledge exchange systems to engage with policymakers could support evidence based policy, more open policy making and joined‐up policies.

## Conflict of Interest

None declared.
